# A Case-Control Study on the Take-Off Sign in Lesional Skin Biopsies of Patients with Pityriasis Rosea

**Published:** 2016

**Authors:** Chuh A, Zawar V, Karad G

**Affiliations:** 1 *JC School of Public Health, The Chinese University of Hong Kong and Prince of Wales Hospital, Shatin, Hong Kong*; 2 *Skin Diseases Center, Nashik, India*

**Keywords:** Acrally-distributed pityriasis rosea, actinic pityriasis rosea, paraviral exanthem, pityriasis rosea of Vidal, viral exanthem

**Dear Editor-in-Chief**

Lesional histopathological features in pityriasis rosea (PR) are non-specific (1). Our aim here is to investigate whether a histopathological take-off sign (TOS) is associated with PR.

We searched our records, and retrieved the 30 most recent patients with PR diagnosed according to a diagnostic criteria (2). Eight records had histopathological images available. For each, we retrieved the next patient record with differential diagnoses of PR (guttate psoriasis, nummular dermatitis, pityriasis versicolor, tinea corporis, drug eruptions) and with histopathological images available.

Six (75%) patients (three with typical PR, one with facial lesions, one with actinic PR, one with PR inversus) and no control subject had TOS (Fig. 1). As we proceed from the uninvolved to the lesional skin (opposite to the direction of the disease progression), the superficial layers of stratum corneum gradually elevate while separating from the more basal layers. The floating part then deviates further away from the specimen, akin to an aircraft taking off.

All controls (one with nummular dermatitis with id eruptions, two with psoriasis vulgaris, one with pityriasis versicolor, two with tinea corporis, one with secondary syphilis, and one with PR-like drug rash) did not exhibit TOS (two-tailed *P*: less than 0.01).

Lesional histopathological changes in PR are non-specific, with inter- and intra-oedema, focal caps of spongiosis (6-9), Unna’s sign (eczematoid pattern), Lowenbach’s sign (thinning of the granular layer), Sabouraud’s sign (exudating erythrocytes in the papillary dermis, homogenisation of papillary collagen (7), and intraepidermal dyskeratotic keratinocytes (4-5) having been reported. However, the sensitivities are low.

The mechanism of TOS is due to peripheral collarette scaling. The morphology of collarette scaling is that fine fragments of scales are attached only at the periphery of the lesion, symbolising a tendency of peeling from the centre towards the edge.

Mysore (2010) reported lifting off parakeratotic scale for PR (10). We believe that the mechanism is related to peripheral collarette scaling and the resultant hanging curtain.

Depression of surface epidermis unrelated to openings of eccrine sweat ducts or pilosebaceous ducts has been reported in lichen sclerosis et atrophicus and PR. We found this sign in two patients, and minimal depression in another two. Further explorations are recommended.

A limitation in our study is the small number of patients. Owing to the nature of the study, we were unable to provide full demographic, clinical, and histological data of the patients.

We thus conclude that PR is significantly associated with TOS.

**Fig. 1:**
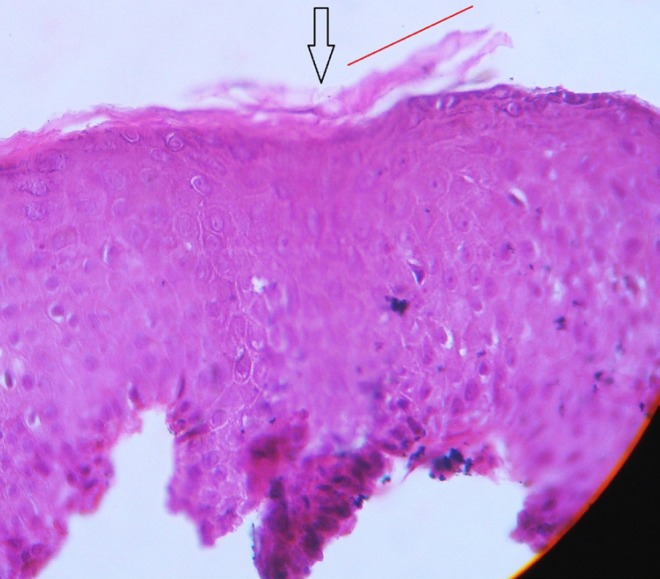
Lesional histopathology at the edge of a lesion of a 14-year-old boy with typical pityriasis rosea. (Black arrow: the point of *take-off*; red line: the direction of *take-off*

## Conflict of Interest:

The authors declare that there is no Conflict of Interests.
